# Just wrong? Or just WEIRD? Investigating the prevalence of moral dumbfounding in non-Western samples

**DOI:** 10.3758/s13421-022-01386-z

**Published:** 2023-01-17

**Authors:** Cillian McHugh, Run Zhang, Tanuja Karnatak, Nishtha Lamba, Olga Khokhlova

**Affiliations:** 1 Department of Psychology, University of Limerick, Limerick, Ireland; 2 SOCOUL Lab, Department of Psychology, University of Limerick, Limerick, Ireland; 3 Centre for Social Issues Research, Department of Psychology, University of Limerick, Limerick, Ireland; 4 Department of Psychology, Middlesex University, Dubai, UAE

**Keywords:** Moral dumbfounding, WEIRD, Moral judgment, China, India, MENA

## Abstract

Moral dumbfounding occurs when people maintain a moral judgment even though they cannot provide a reason for this judgment. Dumbfounded responding may include admitting to not having reasons, or the use of unsupported declarations (“It’s just wrong”) as justification for a judgment. Published evidence for dumbfounding has drawn exclusively on samples of WEIRD backgrounds (Western, educated, industrialized, rich, and democratic), and it remains unclear to what extent the phenomenon is generalizable to other populations. Furthermore, the theoretical implications of moral dumbfounding have been disputed in recent years. In three studies we apply a standardized moral dumbfounding task, and show evidence for moral dumbfounding in a Chinese sample (Study 1, *N* = 165), an Indian sample (Study 2, *N* = 181), and a mixed sample primarily (but not exclusively) from North Africa and the Middle East (MENA region, Study 3, *N* = 264). These findings are consistent with a categorization theories of moral judgment.

**Supplementary Information:**

The online version contains supplementary material available at 10.3758/s13421-022-01386-z.

On 26 January 2016, the Scottish public petitions committee rejected a petition to legalize incest between consenting adults. The petition was unanimously rejected in less than a minute on the grounds that there was “no public interest” in pursuing it further. In rejecting the petition without debate, the substantive issues therein were not addressed. These included claims that Scottish law is discriminatory, infringes on the autonomy of the individual, and is incompatible with the European Convention on Human Rights (see Roffee, 2014). By not addressing these issues, the politicians seemingly maintained a position without providing justification for this position. If an individual member of the committee was questioned on the substantive issues, it is not clear that they would have been able to provide justified reasons to support their judgment, and in some cases may appeal to emotion as justification—indeed, the chair of the committee is reported as describing the idea as “abhorrent” (MacNab, 2016), demonstrating such an appeal to emotion. If pressed, it is possible that a committee member may have even demonstrated a phenomenon in moral psychology known as “moral dumbfounding.”

The phenomenon of moral dumbfounding occurs when people maintain a moral judgment even though they cannot provide a reason in support of this judgment (Haidt et al., 2000; McHugh et al., 2017). While definitions of moral dumbfounding vary, definitions generally refer to (i) maintaining a judgment (ii) in the absence of supporting reasons (for a review of definitions of moral dumbfounding, see McHugh et al., 2017). It typically manifests as an explicit admission of not having reasons, or the use of unsupported declarations (e.g., “It’s just wrong”) as a justification for a judgment. For almost 2 decades, evidence for moral dumbfounding was limited to a single study, unpublished in peer reviewed form, and with a total sample of *N* = 30 (Haidt et al., 2000). This meant that, while the phenomenon was widely discussed in the literature, its existence was not well supported by empirical evidence. Recent work (McHugh et al., 2017) developed methods for eliciting and testing moral dumbfounding, and has provided additional evidence for the existence moral dumbfounding though perhaps it is not as widespread as once assumed (e.g., Royzman et al., 2015; see also McHugh et al., 2020). Despite these developments, current evidence for dumbfounding is limited to samples from WEIRD backgrounds (Western, educated, industrialized, rich, and democratic; see Henrich et al., 2010). Here we address this limitation, and investigate if moral dumbfounding exists beyond these contexts.

## Moral dumbfounding in the literature: Influence and explanations

The discovery of moral dumbfounding (Haidt et al., 2000) coincided with, and arguably contributed to (e.g., Haidt, 2001), a significant shift in theorizing about moral judgment. The classic interpretation of moral dumbfounding is that it provides evidence for the intuitive nature of moral judgments, over more rationalist perspectives (e.g., Haidt, 2001; see also Cushman et al., 2010; Hauser et al., 2008; Prinz, 2005). According to this view, moral judgments are based on intuitions rather than on principles or reasons. Haidt (2001) describes a moral intuition as “the sudden appearance in consciousness of a moral judgment, including an affective valence (good–bad, like–dislike), without any conscious awareness of having gone through steps of searching, weighing evidence, or inferring a conclusion” (p. 818); Prinz (2005) argues that “morally relevant events cause emotional responses” and that to judge something as “morally bad is to recognize an aversive response to it” (p. 99). A key feature of these accounts is the rejection of deliberative reasoned moral judgments in favor of judgments grounded in intuitive or emotional responses. The occurrence of moral dumbfounding supports these intuitionist accounts, whereby people presenting as dumbfounded fail to provide reasons or justifications for their judgments, suggesting their judgments are indeed based on automatic, emotional, or gut reactions. However, while these present a descriptively plausible account of moral judgments, details regarding the specific nature of these responses are unclear, and, beyond a general appeal to emotion, the mechanisms underlying these kind of intuitive moral judgments are not well explained.

The intuitionist perspectives on moral dumbfounding have been contested, with some authors offering theoretical critiques (Dwyer, 2009; Guglielmo, 2018; Jacobson, 2012; Sneddon, 2007; Wielenberg, 2014), while others additionally provide empirical tests of assumptions relevant to dumbfounding (Gray & Keeney, 2015; Gray et al., 2014; Stanley et al., 2019). A central argument in these critiques is that moral judgments are based on reasons or principles (e.g., harm), even in the dumbfounding paradigm. For instance, Royzman et al. (2015) showed that when provided with principles or possible reasons (e.g., harm based/norm based) to justify a judgment, people readily endorsed these reasons. Importantly, when participants who endorsed these options were excluded from analysis Royzman et al. (2015) found that dumbfounding effectively disappeared. This undermines the classic intuitive narrative regarding moral dumbfounding, and has been cited as support for other approaches—for example, theory of dyadic morality (TDM), which posits that all moral judgments, even in cases of moral dumbfounding, are grounded in perceived harm (Schein & Gray, 2018).

Despite these challenges to moral dumbfounding, the phenomenon continues to be observed (though perhaps with a lower incidence than suggested by earlier reports). McHugh et al. (2017, Study 1) replicated the original unpublished interview study demonstrating dumbfounding and developed methods for testing for dumbfounding in larger samples (Studies 2 and 3). McHugh et al., (2020) developed stricter exclusion criteria than those used by Royzman et al. (2015), with demonstrably greater accuracy (evidenced by a large and consistent reduction in the measurable rate of false exclusions; Royzman et al. criteria resulted in a 50% false exclusion rate while the McHugh et al. false exclusion rate was less than 10%). Using these stricter exclusion criteria, McHugh et al. (2020) showed evidence for dumbfounded responding, and that these responses could not be attributed to either harm-based or norm-based reasons (Royzman et al., 2015). This poses a challenge both to the conclusions of Royzman et al. (2015) and to these reason-based explanations more generally.

Over the past 2 decades, dual-process theories of moral judgment (Conway & Gawronski, 2013; Crockett, 2013; Cushman, 2013; Greene, 2008, 2016; Reynolds & Conway, 2018) have emerged as influential approaches to understanding moral judgment. According to these approaches moral judgments are grounded in both deliberative and intuitive processes, these different processes are invoked under different conditions, and lead to different kinds of moral judgments (Greene, 2016). By integrating assumptions of both deliberative and intuitive processes into a unified approach dual-process accounts have alleviated the tension between emotional/intuitionist (e.g., Haidt, 2001; Prinz, 2005) and principle/reason-based accounts (e.g., Royzman et al., 2015). However, on the issue of moral dumbfounding these dual-process theories are relatively silent, and do not offer a clear explanation of how the phenomenon occurs. Thus despite its early influence on theorizing about moral judgment, dumbfounding remains a phenomenon that is poorly understood in the moral psychology literature.

Recent work by McHugh et al. (2022) presents an alternative theoretical perspective that directly addresses the question of moral dumbfounding. According to moral judgment as categorization (MJAC; McHugh et al., 2022), making moral judgments involves the same cognitive processes that underlie categorization more generally; when we make a moral judgment, we *categorize* something as “wrong” or “right” (or indeed “not morally relevant”). These categorizations are dynamical, context dependent, and always occur as part of goal-directed activity. In this view, (relative) stability in moral categorization emerges through continued and consistent repetition and rehearsal in making relevant categorizations as part of goal-directed activity. People to become skilled at making these categorizations such that these categorizations become habitualized or automatic/intuitive (Barsalou, 2017; McHugh et al., 2022).

Barsalou (1999, 2003) describes the process of type-token interpretation as the mechanism through which categorizations are learned and maintained. Type-token interpretation is defined as the binding of specific tokens (category members) to general types (categories), put simply, it is the process of identifying something as a member of a particular category (Barsalou, 2003). Importantly, this does not necessarily entail explicit naming of categories and category members, but often occurs implicitly through our interactions with category members as part of goal-directed activity—that is, simply treating an item as a member of a given category as part of goal-directed activity (McHugh et al., 2022). To illustrate, imagine you are talking to a colleague, Sam. As the conversation develops, Sam begins gossiping about another colleague (Alex), including discussing something that Alex told Sam in confidence. This makes you uncomfortable, and you change the subject. In addition, you resolve to be wary of discussing anything sensitive with Sam in the future. This response did not involve any overt expression of disapproval, or explicitly labeling Sam’s behavior, however the response was sufficient for implicit type-token interpretation to take place. Furthermore, similar implicit type-token interpretation will likely occur during future interactions with Sam as you steer the conversation away from sensitive topics (see McHugh et al., 2022, pp. 133–135 for a more detailed explication of this process in relation to the learning of an ad hoc goal-derived category).

In addition to providing an account for implicit learning of moral categorizations, the categorization approach also allows for the development of automaticity through conscious deliberation (e.g., Pizarro & Bloom, 2003). That is, rules or principles can be learned and applied through deliberative processes, whereby each time the rule is applied to make a relevant categorization type-token interpretation takes place. Automaticity emerges when this type-token interpretation occurs multiple times across different contexts (McHugh et al., 2022; Pizarro & Bloom, 2003). As such, a categorization approach can accommodate diverse approaches including harm-based morality such as theory of dyadic morality (see Schein & Gray, 2018, p. 42), as part of a broader and more diverse, dynamic and context-sensitive understanding of morality (however such an approach rejects claims that harm is the essence of morality, indeed, and such essentialist claims are not supported empirically, e.g., McHugh et al., 2020; Royzman & Borislow, 2022).

A key assumption of MJAC is that moral categorizations are dynamical and subject to a range of contextual influences. Contextual influences known to impact moral judgments include order effects, intentionality, evitability, and, most relevant for the current discussion, emotions (see McHugh et al., 2022 for discussion). According to MJAC, when we interact with particular category member, the specific context of a given interaction matters both for the current interaction, *and* for future interactions. Consider emotional responses, when we encounter a particular moral transgression (e.g., murder, mutilation) we may experience a particular emotion (e.g., shock/anger, disgust). Over time, this may lead specific emotions to become associated with particular moral transgressions, such that the experience of the emotion may serve as a contextual cue that may prompt particular moral categorizations or make this categorization more likely in a given situation (see Barsalou, 2003; Cameron et al., 2013; Damasio, 1994; Giner-Sorolla, 2018).

Regarding the question of moral dumbfounding, we expect that dumbfounding emerges for categorizations that have been learned and maintained primarily through implicit type-token interpretation. Dumbfounding is described as typically occurring for harmless taboo behaviors (Jacobson, 2012). McHugh et al. (2022) note that taboo behaviors do not typically evoke much discussion (recall the case involving the Scottish public petitions committee discussed above; Sim, 2016). As these behaviors are considered taboo, they are also likely to elicit (and therefore be reliably associated with) an emotional response (e.g., disgust, shame, embarrassment). This means that these behaviors are reliably categorized as morally wrong and are often associated with an aversive emotional response; however, they are not associated with any discussion or consideration of the reasons behind this categorization. When people are challenged to identify reasons for their categorization of these behaviors, they may struggle, and in some case present as dumbfounded.

This explanation has two important implications relevant to the current research. First, it suggests that dumbfounding may not be unique to specialized scenarios. While some scenarios (e.g., taboo) may more reliably evoke dumbfounded responding, people may be dumbfounded by any categorization (moral or non-moral) that has become habitualized independently of consideration of the reasons for this categorization. McHugh et al. (2022) discuss the possibility that dumbfounding may be observed beyond the moral domain (e.g., research on the illusion of explanatory depth; Keil et al., 2004; see also Boyd, 1989, 1991; Keil, 1989; we note that while not directly referred to as dumbfounding, there is a strong case for viewing these as equivalent phenomena—see McHugh et al., 2022; it is also apparent that people are generally skilled at rationalizing such that a state of dumbfoundedness can often be successfully avoided, e.g., Haidt, 2001; Jefferson, 2020; Nisbett & Wilson, 1977). Within the moral domain, some previous work has investigated more than one moral scenario (McHugh et al., 2017); however, the majority of published work on moral dumbfounding has focused on a single moral dilemma (an act of consensual incest between a brother and sister; e.g., McHugh et al., 2020; Royzman et al., 2015). Second, explaining dumbfounding as a consequence of domain-general learning processes suggests that dumbfounding is not unique to specific populations. However, current evidence for dumbfounding is based exclusively on studies involving participants from WEIRD countries (Henrich et al., 2010).

Thus, the contribution of the current research is twofold. Firstly, there is a strong empirical case for extending existing research on moral dumbfounding to national contexts not previously studied. Secondly, research on moral dumbfounding continues to inform the development of theories of moral judgment, with MJAC (McHugh et al., 2022) predicting that moral dumbfounding should occur (a) for diverse scenarios and (b) in diverse populations. The current studies offer a preliminary test of both these predictions.

## The current research

In three studies, we test the incidence of moral dumbfounding in non-Western samples, using several moral scenarios. In Study 1, we assess whether or not moral dumbfounding can be elicited in a Chinese sample. In Study 2, we investigate whether or not moral dumbfounding can be found in an Indian sample. In Study 3, we test for moral dumbfounding in a sample primarily (but not exclusively) from MENA (Middle East and North Africa) region.

Given that this is the first study of moral dumbfounding in a non-Western setting, we additionally investigated the potential influence of culturally relevant individual differences on moral dumbfounding. A measure that is widely regarded as one of the most prominent dimensions that varies with culture is individualism/collectivism (Renzhi et al., 2013), therefore we included the individualism-collectivism scale (ICS: Li & Aksoy, 2007; Renzhi et al., 2013; Triandis & Gelfand, 2011) for exploratory purposes.

In each study (with the exception of Study 1b), we presented participants with four scenarios. Given the tendency for patterns of responding to vary across scenarios (McHugh et al., 2017), our primary analyses focus on the different scenarios separately. However, previous work has shown interindividual variability in patterns of responding to specific scenarios (McHugh et al., 2017). As such, for each study below we additionally provide an overall estimate of incidence of dumbfounding within the sample—that is, we report the total number of participants who provided a dumbfounded response at least once across the four scenarios.

## Study 1: Chinese sample

The aim of Study 1 was to investigate if, and how, moral dumbfounding is elicited in a Chinese sample. Furthermore, we measure individual differences in individualism/collectivism and test for a possible relationship between these dimensions and dumbfounded responding.

### Method

#### Participants and design

Study 1 was a frequency-based design, using the methods and materials developed by McHugh et al. (2017), to test whether dumbfounded responding could be evoked in a Chinese context. Results are primarily descriptive. We include exploratory analyses investigating the possible influence of individualism/collectivism (Triandis & Gelfand, 2011) on responding.

A total of 165 individuals participated. Our sample sizes in all studies are informed by previous research (McHugh et al., 2017; Royzman et al., 2015). Participants were undergraduate and postgraduate students at Luoyang Normal University (China). All participants were Chinese citizens residing in China at the time of completing the study. One part of the sample, including 42 participants (34 female, eight male, zero other; *M*_age_ = 21.43 years, min = 18, max = 27, *SD* = 1.74), completed four scenarios, described in the next section. Another part of the sample, including 123 participants (75 female, 48 male, zero other; *M*_age_ = 22.06 years, min = 18, max = 45, *SD* = 3.76) completed only the *Cannibal* scenario (for clarity, these studies are reported separately as Study 1a and Study 1b). Participation was voluntary and participants were not reimbursed for their participation.

#### Procedure and materials

Data were collected through the Chinese language online survey software Wenjuanxing (Changsha Ranxing Information Technology Co., Ltd., 2006). Participants were provided with a link to the online survey, containing an information sheet and a consent form. Participants could only proceed to the remainder of the survey if they provided consent. Next, participants were presented with demographic questions.

The procedure and materials for the moral dumbfounding task were taken directly from McHugh et al. (2017). These were translated into Chinese by a member of the research team whose native language was Chinese. Back translation methodology was used to ensure that the original meaning of the content was not compromised and that the scales were culturally adaptive (Brislin, 1970). Four moral judgment scenarios were used, loosely identified as “intuition” scenarios: *Incest* and *Cannibal*; and “reasoning” scenarios: *Trolley* and *Heinz* (taken from McHugh et al., 2017). *Incest* describes an act of consensual incest between a brother and sister using contraception; *Cannibal* describes a research assistant in a pathology lab who takes home and eats meat from a cadaver due to be discarded; in *Trolley*, a character pushes someone from a bridge to stop a runaway trolley heading towards five people (killing one to save five); *Heinz* describes a man stealing drugs to save his wife (see Appendix A for full text of all scenarios).

#### Moral dumbfounding task

Participants are presented with a scenario to read. They were asked to rate, on a 7-point Likert scale (1 = *morally wrong*; 4 = *neutral*; 7 = *morally right*), how right or wrong they regarded the behavior described in the scenario. Following this, participants were asked to rate their confidence in their judgment (again on a 7-point Likert scale, 1 = *extremely doubtful*; 4 = *neutral*; 7 = *extremely confident*). Participants are then presented with a series of counterarguments, which refuted commonly used justifications for rating the behaviors as “wrong” (see Appendix B). After each counterargument, participants are asked “Do you (still) think it is wrong?” with a binary “yes/no” response option; and then they are asked “Do you have a reason for your judgment?” with three possible response options: “Yes, I have a reason,” “No, I have no reason,” and “Unsure.” This sequence was repeated for each of the three counterarguments.

Dumbfounding is measured using the “critical slide,” which contains a statement defending the behavior, and a question asking how the behavior could be wrong (see Appendix C). There are three possible answer options: (a) “There is nothing wrong”; (b) an admission of not having reasons (“It’s wrong but I can’t think of a reason”); and finally a judgment with accompanying justification (c) “It’s wrong and I can provide a valid reason.” The selecting of option (b), the admission of not having reasons, is taken to be a dumbfounded response. Participants who selected (c) were promoted to type a reason on the next page. The order of these response options was randomized.

Following the critical slide, participants rated the behavior again, and completed the postdiscussion questionnaire devised by Haidt et al. (2000). They were required to rate on a 7-point Likert scale how sure they were about their judgment; how much they changed their mind; how confused and how irritated they were; to what extent their judgment was based on reason, and to what extent on “gut” feeling (see Appendix D). This process is repeated for each moral scenario. The order of presentation of the moral scenarios was randomized.

#### Coding reasons

While there is a strong theoretical and empirical case for coding the reasons provided for unsupported declarations or tautological responses, as dumbfounded responses (see McHugh et al., 2017), this approach has been challenged by claims that these responses constitute the expression of a normative position (e.g., Royzman et al., 2015). In response to this challenge, we adopt an “admission of not having reasons” as the only measure of moral dumbfounding in these studies. While this measure provides a more conservative estimate of the prevalence of dumbfounding, it has higher face validity and provides a less ambiguous estimate.

#### Individualism-collectivism scale

Following the dumbfounding task, participants completed the individualism-collectivism scale (Renzhi et al., 2013; see Appendix E). This 16-item scale includes four subscales: Vertical Collectivism (VC), Horizontal Collectivism (HC), Vertical Individualism (VI), and Horizontal Individualism (HI). Collectivism is characterized by common goals, interpersonal relationships, social dependencies, and connections. Individualism emphasizes individual goals and independence. Horizontal refers to egalitarianism, while vertical emphasizes authority, principles, and hierarchy (Triandis & Gelfand, 2011). Regarding the specific combinations of these dimensions, VC maintains the authoritative structure within the organization, supporting self-sacrifice and competition outside the organization. In addition to treating the self as part of the organization, HC also emphasizes the equality of members within the group. VI means the increase of achievement based on individualism, with emphasis on independence and placing the self on any interpersonal relationship. HI refers to the addition of universal values based on individualism, and independence is to maintain a certain meaning or freedom within a principle (Triandis & Gelfand, 2011). The responses were recorded on a 9-point Likert scale, ranging from 1 = *strongly disagree* to 9 = *strongly agree*. The reliabilities for the four subscales are as follows, Study 1a: α_VI_ = .59; α_VC_ = .53; α_HC_ = .42; α_HI_ = .81; Study 1b: α_VI_ = .53; α_VC_ = .50; α_HC_ = .55; α_HI_ = .67. The entire study lasted approximately 20 minutes.

### Results

#### Judgments of the scenarios

The proportion of wrong, neutral, and OK, judgments are displayed in Table 1. Descriptive statistics for judgment, confidence, and changed-mind ratings are displayed in Table 2, with descriptive statistics for the postdiscussion questionnaire displayed in Table 3.

**Table 1:** Valence of initial and revised judgments for each scenario for each study

		Heinz		Trolley		Incest/Promise		Cannibal/Dog	
		*N*	percent	*N*	percent	*N*	percent	*N*	percent
Study 1a	Initial: Wrong	9	21.43%	17	40.48%	25	59.52%	37	88.1%
	Initial: Neutral	13	30.95%	19	45.24%	8	19.05%	4	9.52%
	Initial: OK	20	47.62%	6	14.29%	9	21.43%	1	2.38%
	Revised: Wrong	10	23.81%	19	45.24%	23	54.76%	37	88.1%
	Revised: Neutral	12	28.57%	14	33.33%	10	23.81%	5	11.9%
	Revised: OK	20	47.62%	9	21.43%	9	21.43%	0	0%
Study 1b	Initial: Wrong	–	–	–	–	–	–	84	68.29%
	Initial: Neutral	–	–	–	–	–	–	21	17.07%
	Initial: OK	–	–	–	–	–	–	18	14.63%
	Revised: Wrong	–	–	–	–	–	–	80	65.04%
	Revised: Neutral	–	–	–	–	–	–	28	22.76%
	Revised: OK	–	–	–	–	–	–	15	12.2%
Study 2	Initial: Wrong	130	71.82%	125	69.06%	115	63.54%	144	79.56%
	Initial: Neutral	16	8.84%	18	9.94%	44	24.31%	27	14.92%
	Initial: OK	35	19.34%	38	20.99%	22	12.15%	10	5.52%
	Revised: Wrong	138	76.24%	123	67.96%	109	60.22%	145	80.11%
	Revised: Neutral	12	6.63%	22	12.15%	37	20.44%	24	13.26%
	Revised: OK	31	17.13%	36	19.89%	35	19.34%	12	6.63%
Study 3	Initial: Wrong	147	58.1%	142	56.13%	75	29.64%	162	64.03%
	Initial: Neutral	33	13.04%	38	15.02%	93	36.76%	30	11.86%
	Initial: OK	35	13.83%	33	13.04%	60	23.72%	23	9.09%
	Revised: Wrong	139	54.94%	130	51.38%	47	18.58%	158	62.45%
	Revised: Neutral	35	13.83%	48	18.97%	91	35.97%	29	11.46%
	Revised: OK	36	14.23%	32	12.65%	85	33.6%	23	9.09%

Study1a and 1b are Chinese samples, Study 2 is an Indian sample, Study 3 sample is primarily from MENA

**Table 2:** Means and Standard Deviations for initial and revised confidence ratings, and for self-reported changed mind for each scenario for each study

		Heinz		Trolley		Incest/Promise		Cannibal/Dog	
		*M*	*SD*	*M*	*SD*	*M*	*SD*	*M*	*SD*
Study 1a	Initial Judgment	4.8	2.1	3.3	1.9	2.9	2.2	1.5	1.1
	Revised Judgment	4.7	2.0	3.4	2.0	2.9	2.1	1.6	1.1
	Initial Confidence	5.5	1.3	5.5	1.5	5.4	1.2	5.8	1.7
	Revised Confidence	5.7	1.4	5.4	1.7	5.4	1.5	6.1	1.5
	Changed Mind	2.7	2	2.6	1.9	2.5	2.1	2.5	2.1
Study 1b	Initial Judgment	–	–	–	–	–	–	2.5	2.1
	Revised Judgment	–	–	–	–	–	–	2.5	2.0
	Initial Confidence	–	–	–	–	–	–	4.9	1.6
	Revised Confidence	–	–	–	–	–	–	5.1	1.7
	Changed Mind	–	–	–	–	–	–	2.5	1.7
Study 2	Initial Judgment	2.6	1.9	2.8	1.8	2.6	1.8	2.1	1.5
	Revised Judgment	2.6	1.8	2.8	1.8	2.9	1.9	2.1	1.4
	Initial Confidence	5.9	1.2	5.3	1.6	5.8	1.4	5.6	1.4
	Revised Confidence	5.8	1.3	5.5	1.5	5.7	1.4	5.7	1.3
	Changed Mind	1.7	1.3	2.1	1.5	2	1.6	1.8	1.4
Study 3	Initial Judgment	2.7	1.8	2.6	1.9	3.9	1.7	2.2	1.8
	Revised Judgment	2.9	1.7	2.9	1.9	4.4	1.6	2.3	1.8
	Initial Confidence	5.8	1.6	5.4	1.8	5.6	1.5	5.8	1.6
	Revised Confidence	5.6	1.6	5.5	1.6	5.7	1.4	6	1.5
	Changed Mind	2.1	1.6	2.1	1.5	2.2	1.7	1.7	1.3

Anchors for all Likert items range from 1 to 7 (Judgments: 1 = *morally wrong*, 7 = *morally right*; Confidence: 1 = *extremely doubtful*, 7 = *extremely confident*; Changed Mind: 1 = *not at all*, 7 = *extremely*)

**Table 3:** Means and standard deviations for responses to the postdiscussion questionnaire for each scenario for each study

		Heinz		Trolley		Incest/Promise		Cannibal/Dog	
		*M*	*SD*	*M*	*SD*	*M*	*SD*	*M*	*SD*
Study 1a	Confused	2.3	1.6	3	2.2	2.3	1.6	2.4	2.1
	Irritated	2.3	1.6	2.9	2	2.5	2	3.6	2.5
	Reason-Based	5.2	1.8	5.2	1.8	5.6	1.6	5.6	1.8
	Gut-Based	3.5	2	3.7	2	3.4	2	3.7	2.3
Study 1b	Confused	–	–	–	–	–	–	3	2.1
	Irritated	–	–	–	–	–	–	3.7	2.1
	Reason-Based	–	–	–	–	–	–	5.2	1.6
	Gut-Based	–	–	–	–	–	–	4.3	1.9
Study 2	Confused	1.9	1.4	2.4	1.7	2	1.6	2	1.4
	Irritated	2.2	1.7	2.3	1.7	2.4	1.9	2.6	1.9
	Reason-Based	5.5	1.7	5.1	1.8	5.1	1.8	5.2	1.9
	Gut-Based	3.5	2.3	3.4	2.1	3.8	2.2	3.4	2.2
Study 3	Confused	2.4	1.9	2.8	2	2.3	1.8	2.1	1.7
	Irritated	3.3	2.3	3.3	2.2	2.3	1.8	4	2.4
	Reason-Based	5.5	1.5	5.4	1.8	5.5	1.7	5.5	1.8
	Gut-Based	3.9	2.1	3.7	2.2	3.9	2.2	4	2.4

Anchors for all Likert items range from 1 to 7 (1 = *not at all*, 7 = *extremely*)

A paired-samples *t* test revealed no differences in judgment ratings from time one to time two, *Heinz* (*p* = .958); *Cannibal* (*p* = .768); *Incest* (*p* = .960); *Trolley* (*p* = .870).

A one-way analysis of variance (ANOVA) revealed significant differences in initial judgments depending on scenario, *F*(3, 164) = 20.77, *p* < .001, partial η^2^ = .275. Tukey’s post hoc pairwise comparison revealed that judgments in the *Heinz* dilemma were significantly more favorable than for each of the other scenarios: *Cannibal*, *p* < .001, *Incest*, *p* < .001, *Trolley*, *p* = .003; while judgments of *Cannibal* were significantly more harsh than all other scenarios: *Heinz*, *p* < .001, *Incest*, *p* = .007. *Trolley*, *p* < .001; there was no significant difference between initial judgments of *Incest* and of *Trolley*, *p* = .762.

A one-way ANOVA revealed the same pattern of differences in revised judgments depending on scenario, *F*(3, 164) = 20.19, *p* < .001, partial η^2^ = .270. Again, Tukey’s post hoc pairwise comparison revealed that judgments in the *Heinz* dilemma were significantly more favorable than for each of the other scenarios: *Cannibal*, *p* < .001, *Incest*, *p* < .001, *Trolley*, *p* = .005; while judgments of *Cannibal* were significantly harsher than all other scenarios: *Heinz*, *p* < .001, *Incest*, *p* = .009. *Trolley*, *p* < .001; there was no significant difference between revised judgments of *Incest* and of *Trolley*, *p* = .685.

#### Measuring dumbfounding

**Fig. 1:**
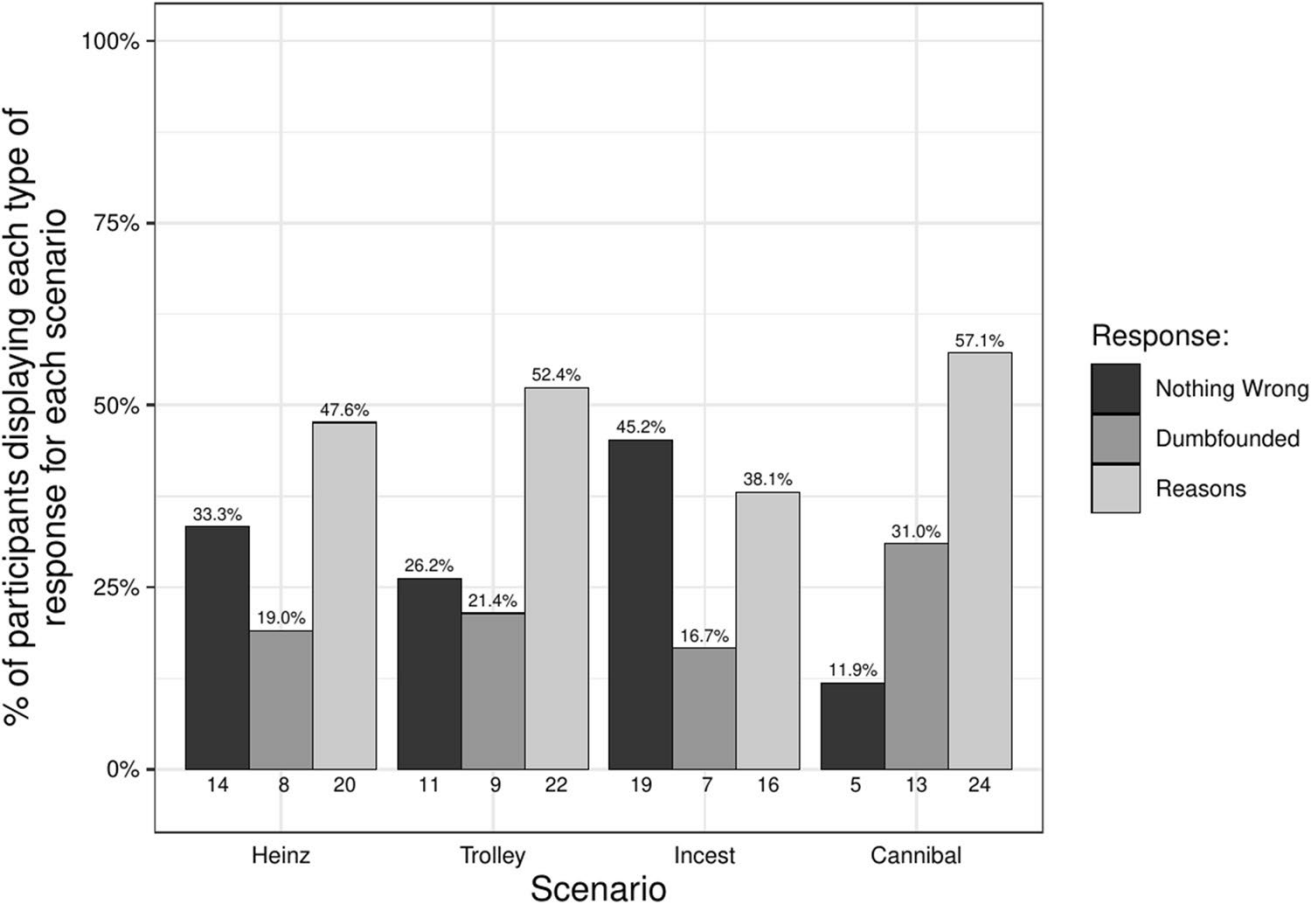
Rates of each type of response for each scenario in the Chinese sample (*N* = 42)

**Fig. 2:**
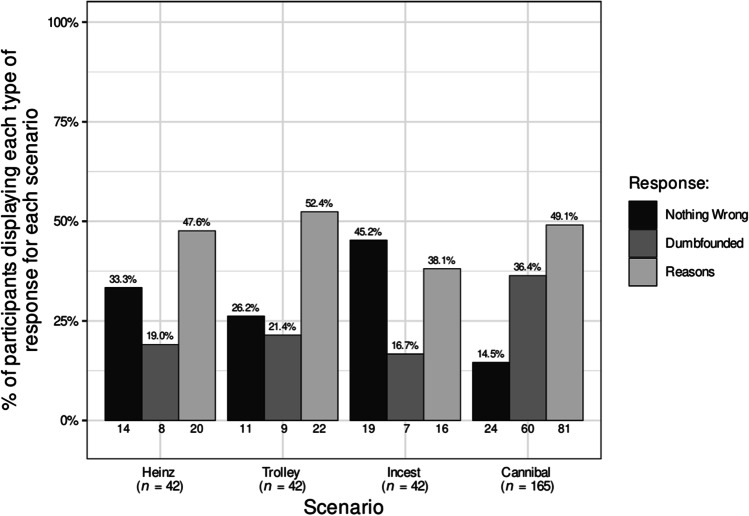
Rates of each type of response for each scenario in the Chinese sample (including additional data on Cannibal scenario)

Participants who selected the admission of not having reasons were identified as dumbfounded. Across the four scenarios (Study 1a), 21 participants (50%) provided a dumbfounded response at least once. In Study 1b, 47 participants, (38.21%) provided a dumbfounded response for the *Cannibal* scenario. Table 4 shows the number and percentage of participants who selected each response for each scenario across Studies 1a and 1b. Figure 1 shows this information for Study 1a, while Fig. 2 additionally includes the responses for Study1b.

**Table 4:** Observed frequency and percentage of each of the responses: dumbfounded, nothing wrong, and reasons provided for each scenario for each study

		Heinz		Trolley		Incest/Promise		Cannibal/Dog	
		*N*	percent	*N*	percent	*N*	percent	*N*	percent
Study 1a	Nothing wrong	14	33.33%	11	26.19%	19	45.24%	5	11.9%
	Dumbfounded	8	19.05%	9	21.43%	7	16.67%	13	30.95%
	Reasons	20	47.62%	22	52.38%	16	38.1%	24	57.14%
Study 1b	Nothing wrong	–	–	–	–	–	–	19	15.45%
	Dumbfounded	–	–	–	–	–	–	47	38.21%
	Reasons	–	–	–	–	–	–	57	46.34%
Study 2	Nothing wrong	49	27.07%	41	22.65%	72	39.78%	33	18.23%
	Dumbfounded	20	11.05%	47	25.97%	33	18.23%	44	24.31%
	Reasons	112	61.88%	93	51.38%	76	41.99%	104	57.46%
Study 3	Nothing wrong	58	22.92%	36	14.23%	159	62.85%	44	17.39%
	Dumbfounded	30	11.86%	48	18.97%	22	8.7%	41	16.21%
	Reasons	123	48.62%	126	49.8%	44	17.39%	130	51.38%

A series of *z* tests indicated that rates of dumbfounded responding for each scenario in Study 1a were significantly greater than zero, *Heinz*: *z* = 2.97, *p* = .003; *Trolley*: *z* = 3.17, *p* = .001; *Incest*: *z* = 2.76, *p* = .006; *Cannibal*: *z* = 3.92, *p* < .001. Similarly rates of dumbfounded responding in Study 1b were significantly greater than zero for the *Cannibal* scenario, *z* = 7.62, *p* < .001.

There was no significant difference in observed rates of dumbfounded responding depending on which scenario was being discussed, χ^2^(6, *N* = 253) = 12.34, *p* = .055. Similarly, there was no influence of type of scenario (reasoning vs. intuition) on rates of dumbfounded responding χ^2^(2, *N* = 253) = 0.31, *p* = .855.

#### Individual differences

A hierarchical linear regression was conducted to test the possible relationship between ICS (Renzhi et al., 2013) and susceptibility to dumbfounding. Susceptibility to dumbfounding was operationalized by creating a new variable representing the number of times each participant provided a dumbfounded response across the four scenarios. This measure was included as our outcome variable, and the four subscales of ICS were included as predictor variables. The overall model did not significantly predict susceptibility to dumbfounding, *R*^2^ = .09, *F*(4, 38) = 0.95, *p* = .488.

We conducted a series of multinomial logistic regressions to investigate the possible relationship between ICS (Renzhi et al., 2013) and responding to each of the scenarios individually. Response to the critical slide scenario was the dependent variable for each scenario, and the four subscales of the ICS were included as predictor variables.

The overall model did not significantly predict responses for any of the scenarios in Study 1a (*Heinz*, *p* = .233; *Trolley*, *p* = .201; *Incest*, *p* = .084; *Cannibal*, *p* = .554). Similarly, in Study 1b, the overall model did not significantly predict responses to the critical slide for the *Cannibal* scenario (*p* = .204).

## Study 2: Indian sample

Having demonstrated dumbfounded responding in a Chinese context, the aim of Study 2 was to assess if dumbfounded responding can be elicited in an Indian context. Furthermore, we introduced an additional individual difference variable. Previous work has indicated a possible link between meaning and morality (e.g., Bellin, 2012; Schnell, 2011), and as such, we included the Meaning in Life Questionnaire (MLQ; Steger et al., 2008) in Study 2.

### Method

#### Participants and design

Study 2 was a frequency-based design using the methods and materials developed by McHugh et al. (2017). The aim of Study 2 was to identify whether dumbfounded responding could be evoked in an Indian context.

A total sample of 181 (114 female, 64 male, zero other, three declined to report their gender; *M*_age_ = 22.96, min = 18, max = 39, *SD*_age_ = 2.42) participants took part. As in Study 1, our sample size was based on resources available and on previous research. The breakdown of participants’ religion is as follows, Hinduism: *n* = 133, Islam: *n* = 4, Christianity: *n* = 7, Sikhism: *n* = 3, Buddhism: *n* = 0, Jainism: *n* = 8, other: *n* = 9, and 17 participants declined to provide information about their religion. All participants were of Indian nationality, and 158 indicated that they resided in India at the time of completing the survey. Participants were recruited through convenience and snowball sampling.

#### Procedure and materials

The procedure for Study 2 was the same as Study 1, with some minor changes. Given the diversity of languages in India, and the high proficiency of English among Indian nationals, all materials were presented in English. The survey was presented using Qualtrics. The demographic information recorded included religion, given the prominence and diversity of religions in Indian society.

The reliabilities for the ICS subscales are: α_VC_ = .78; α_VI_ = .55; α_HC_ = .70; α_HI_ = .78. We also included the Meaning in Life Questionnaire (MLQ; Steger et al., 2008). The reliabilities for the subscales of the MLQ are as follows: α_Presence_ = .81; α_Search_ = .80. The study lasted 20–25 minutes.

### Results

#### Judgments of the scenarios

The proportion of wrong, neutral, and OK judgments for each scenario are displayed in Table 1. Descriptive statistics for judgment, confidence, and changed-mind ratings are in Table 2, and postdiscussion questionnaire results are in Table 3.

A paired-samples *t* test revealed no differences in judgment ratings from time one to time two for any of the scenarios (*Heinz*, *p* = .842; *Cannibal*, *p* = .772; *Incest*, *p* = .162; *Trolley*, *p* = .977).

A one-way ANOVA revealed significant differences in initial judgments depending on scenario, *F*(3, 720) = 6.14, *p* < .001, partial η^2^ = .025. Tukey’s post hoc pairwise comparison revealed that judgments of *Cannibal* were significantly harsher than all other scenarios: *Heinz*, *p* = .026, *Incest*, *p* = .010. *Trolley*, *p* < .001; there were no significant differences in the ratings of the other scenarios, *Heinz*/*Incest*, *p* = .991, *Heinz*/*Trolley*, *p* = .589, *Incest*/*Trolley*, *p* = .773.

A one-way ANOVA revealed the same pattern of differences in revised judgments depending on scenario, *F*(3, 720) = 7.61, *p* < .001, partial η^2^ = .031. Again, Tukey’s post hoc pairwise comparison revealed that judgments of *Cannibal* were significantly harsher than all other scenarios: *Heinz*, *p* = .028, *Incest*, *p* < .001. *Trolley*, *p* < .001; there were no significant differences in the ratings of the other scenarios, *Heinz*/*Incest*, *p* = .387, *Heinz*/*Trolley*, *p* = .703, *Incest*/*Trolley*, *p* = .957.

#### Measuring dumbfounding

**Fig. 3:**
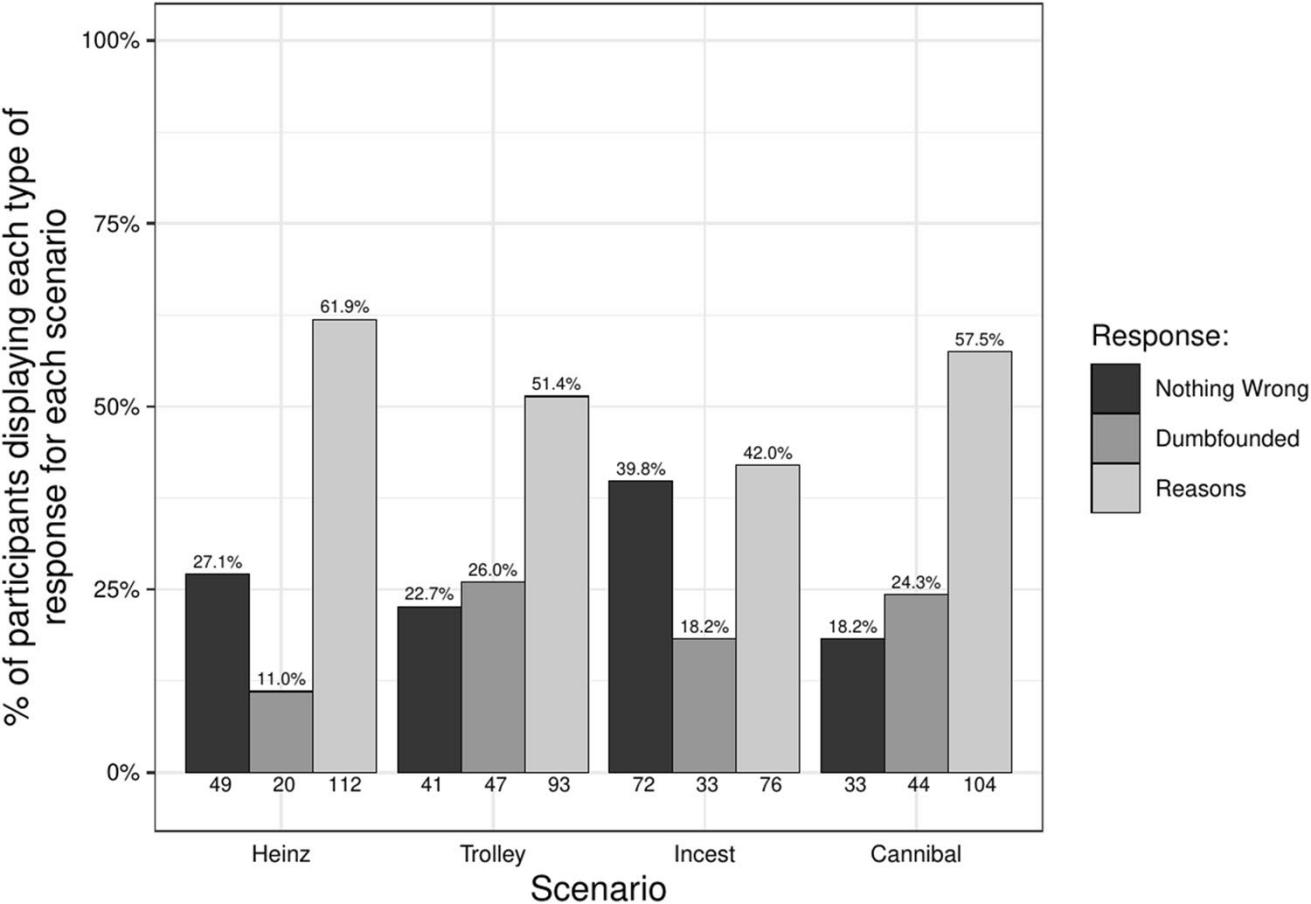
Rates of each type of response for each scenario in the Indian sample (*N* = 181)

Participants selecting the admission of not having reasons were identified as dumbfounded. Across the four scenarios 89 participants (49.17%) provided a dumbfounded response at least once. Table 4 and Fig. 3 show the number and percentage of participants who selected each response for each scenario. Rates of dumbfounded responding for each scenario in Study 2 were significantly greater than zero, *Heinz*: *z* = 4.60, *p* < .001; *Trolley*: *z* = 7.35, *p* < .001; *Incest*: *z* = 6.03, *p* < .001; *Cannibal*: *z* = 7.08, *p* < .001, thus providing evidence for moral dumbfounding in our Indian sample.

A chi-square test for independence revealed significant differences in responses to the critical slide depending on which scenario was being discussed in Study 2, χ^2^(6, *N* = 181) = 37.48, *p* < .001. Table 5 shows the observed counts, expected counts, and standardized residuals for each response for each scenario. For *Heinz*, people were significantly better at providing reasons, and significantly less likely to present as dumbfounded; while people were significantly more likely to be dumbfounded by *Trolley* than expected; for *Incest*, people were significantly less likely to provide reasons, and significantly more likely to select “There is nothing wrong” than expected; finally for *Cannibal* significantly fewer than expected selected “There is nothing wrong.”

**Table 5:** Observed counts, expected counts, and standardized residuals for each response to the critical slide depending on scenario

Response		Heinz	Trolley	Incest	Cannibal
Nothing Wrong	Observed count	49	41	72	33
	Expected count	48.75	48.75	48.75	48.75
	Standardized residuals	0.05	−1.5	4.5**	−3.05*
Dumbfounded	Observed count	20	47	33	44
	Expected count	36	36	36	36
	Standardized residuals	−3.44**	2.37*	−0.65	1.72
Reason	Observed count	112	93	76	104
	Expected count	96.25	96.25	96.25	96.25
	Standardized residuals	2.71*	−0.56	−3.48**	1.33

*p < .05. **p < .001

The observed variability was not related to the type of scenario (intuition vs. reasoning), with no relationship between type of scenario and response to the critical slide being observed, χ^2^(2, *N* = 181) = 3.47, *p* = .176. We additionally tested for the possibility of order effects, and found no significant differences in responses to the critical slide depending on order of presentation χ^2^(6, *N* = 183) = 7.997, *p* = .238.

#### Individual differences

A hierarchical linear regression was conducted to test the possible relationship between ICS (Triandis, & Gelfand, 2011), MLQ (Steger et al., 2008), and susceptibility to dumbfounding. As in Study 1a, we created a new variable by calculating the number of times each participant provided a dumbfounded response, and used this variable as a measure of participants’ susceptibility to dumbfounding. This measure was our outcome variable, and the four subscales of ICS, along with both subscales of the MLQ, were included as predictor variables. The overall model was a significant predictor of susceptibility to dumbfounding *R*^2^ = .07, *F*(6, 174) = 2.29, *p* = .037, with VI as the only variable making a significant contribution to the model, *b* = −0.02, 95% CI [−0.05, 0.00], *t*(174) = −2.02, *p* = .045 (see Table 6).

**Table 6:** Study 2: Predictors of susceptibility to moral dumbfounding

Predictor	*b*	95% CI	*t*(174)	*p*
Intercept	1.60	[0.48, 2.72]	2.82	.005
VC	0.02	[0.00, 0.05]	1.96	.051
HC	0.00	[−0.04, 0.03]	−0.20	.846
VI	−0.02	[−0.05, 0.00]	−2.02	.045
HI	−0.03	[−0.06, 0.00]	−1.70	.090
MLQ Presence	−0.01	[−0.04, 0.01]	−1.12	.263
MLQ Search	0.01	[−0.02, 0.05]	0.93	.352

We conducted a series of multinomial logistic regressions to investigate the possible relationship between ICS (Triandis & Gelfand, 2011) and responding to each of the scenarios individually. Response to the critical slide was the dependent variable for each scenario, and the four subscales of the ICS were included as predictor variables.

The overall model did not significantly predict responses for the *Heinz* dilemma, χ^2^(12, *N* = 181) = 13.48, *p* = .335, the observed power was 0.67; neither did the model significantly predict responses for the *Trolley* scenario, χ^2^(12, *N* = 181) = 12.15, *p* = .433, the observed power was 0.61.

Interestingly, the overall model significantly predicted responses for the *Incest* scenario, χ^2^(12, *N* = 181) = 26.05, *p* = .011, the observed power was 0.95, explaining between 10.69% (Cox and Snell *R* squared) and 14.45% (Nagelkerke’s *R* squared) of the variance in responses to the critical slide. The only significant predictors in the model were HI and VC. As HI increased, participants were significantly more likely to select “there is nothing wrong” than to provide reasons for their judgment, Wald = 7.29, *p* = .007, odds ratio = 1.11, 95% CI [1.03, 1.20]. As VC increased, participants were significantly less likely to present as dumbfounded than to provide reasons, Wald = 4.93, *p* = .026, odds ratio = 0.92, 95% CI [0.85, 1.00].

The overall model also significantly predicted responses for the *Cannibal* scenario, χ^2^(12, *N* = 181) = 23.22, *p* = .026, the observed power was 0.92, explaining between 6.42% (Cox and Snell *R* squared) and 10.47% (Nagelkerke’s *R* squared) of the variance in responses to the critical slide. Meaning in Life: Presence (Steger et al., 2008) was the only significant predictor in the model, as Meaning in Life: Presence, increased, participants were significantly less likely to present as dumbfounded than to provide reasons, Wald = 5.96, *p* = .015, odds ratio = 0.92, 95% CI [0.87, 0.98].

## Study 3: Primarily MENA sample

Having demonstrated dumbfounded responding in targeted samples in two different countries, the aim of Study 3 was to investigate if dumbfounded responding could be elicited in a more diverse sample recruited from a range of non-Western countries.

### Method

#### Participants and design

Study 3 was a frequency based attempted replication of McHugh et al. (2017). The aim of Study 3 was to identify if dumbfounded responding could be evoked in a mixed sample of participants from a selection of non-Western countries, primarily North Africa and the Middle East.

An initial sample of 463 participants were recruited for Study 3. Some participants did not provide full responses for all four scenarios (the total number of participants who completed the critical slide for all four scenarios was *n* = 192). In removing participants with missing data, we retained all participants who completed the critical slide for at least one scenario. Following this, we were left with a total sample of *N* = 264 (160 female, 97 male, three other, four declined to report their gender; *M*_age_ = 28, min = 18, max = 68, *SD*_age_ = 12.68). As in Studies 1 and 2, our sample size was based on resources available and on previous research.

The countries represented in our sample are as follows, Algeria (*n =* 2), Bahrain (*n =* 5), Bangladesh (*n =* 2), Egypt (*n =* 25), India (*n =* 21), Iran (*n =* 2), Iraq (*n =* 9), Israel (*n =* 1), Jordan (*n =* 9), Kuwait (*n =* 5), Lebanon (*n =* 31), Libya (*n =* 14), Morocco (*n =* 1), Oman (*n =* 1), Pakistan (*n =* 8), Palestine (*n =* 14), Philippines (*n =* 13), Saudi Arabia (*n =* 1), South Africa (*n =* 1), Sri Lanka (*n =* 3), Sudan (*n =* 33), Syria (*n =* 30), UAE (*n =* 21), Yemen (*n* = 1).

Our target sample was participants from non-Western countries. As such we removed 11 participants who reported being from Western countries, UK (*n* = 3), USA (*n* = 1), Canada (*n* = 2), Germany (*n* = 1), Portugal (*n* = 1), Netherlands (*n* = 1), and participants who did not provide a country of origin (*n* = 2). This left a total sample of *N* = 253 (154 female, 92 male, three other, four declined to report their gender; *M*_ag*e*_ = 28.05, min = 18, max = 68, *SD*_age_ = 12.54). The breakdown of participants’ religions is as follows, Islam: *n* = 168, Christianity: *n* = 46, Hinduism: *n* = 7, other: *n* = 20, and 12 participants declined to provide their religion.

#### Procedure and materials

The procedure for Study 3 was largely the same as Study 2, with some minor changes. Data collection was conducted in collaboration with colleagues at the Department of Psychology, Middlesex University, Dubai, and participants were recruited through opportunity and snowball sampling by undergraduate students. Given the potentially sensitive and offensive nature of some of the traditional dumbfounding scenarios (*Incest* and *Cannibal*), we replaced these scenarios with scenarios less likely to cause offense: *Promise* and *Dog* (taken from Haidt et al., 1993). *Promise* describes a man who promises his dying mother he will visit her grave every week, but does not keep this promise; in *Dog*, a family’s dog is killed by a car, and the family cook and eat the meat from the dog (for full text, see Appendix A).

The survey was programmed and presented using Qualtrics. The demographic information recorded additionally included participants’ nationality. We also included a filter question in an attempt to limit responses of participants from WEIRD countries. As in Study 2, we also included the ICS (Triandis & Gelfand, 2011), and MLQ (Steger et al., 2008). The reliabilities for the four subscales of the ICS are as follows: VC, α = .80; VI, α = .72; HC, α = .76; HI, α = .80. The reliabilities for the subscales of the MLQ are as follows: MLQ: Presence, α = .90; MLQ: Search, α = .81. The entire study lasted 20–25 minutes.

### Results

#### Judgments of the scenarios

The proportion of wrong, neutral, and OK, judgments for each scenario are displayed in Table 1. Table 2 displays descriptive statistics for judgment, confidence, and changed-mind ratings, while Table 3 displays results for the postdiscussion questionnaire.

A paired-samples *t* test revealed no differences in judgment ratings from time one to time two for *Heinz*, *t*(423.00) = −1.04, *p* = .300, *d* = 0.10; *Dog*, *t*(422.39) = −0.40, *p* = .687, *d* = 0.04; or *Trolley*, *t*(420.79) = −1.57, *p* = .116, *d* = 0.15. In contrast, participants revised ratings of *Promise* (*M* = 4.38, *SD* = 1.64) were significantly more favorable than their initial ratings *M* = 3.92, *SD* = 1.66, *t*(448.94) = −2.99, *p* = .003, *d* = 0.28

A one-way ANOVA revealed significant differences in initial judgments depending on scenario, *F*(3, 867) = 39.15, *p* < .001, partial η^2^ = .119. Tukey’s post hoc pairwise comparison revealed that judgments of *Promise* were significantly more favorable than all other scenarios: *Heinz*, *p* < .001, *Dog*, *p* < .001. *Trolley*, *p* < .001; *Heinz* was rated significantly more favorably than *Dog*, = .017 there were no significant differences in the ratings of the other scenarios, *Heinz*/*Trolley*, *p* = .927, *Dog*/*Trolley*, *p* = .094.

A one-way ANOVA revealed a similar pattern of differences in revised judgments depending on scenario, *F*(3, 849) = 56.42, *p* < .001, partial η^2^ = .166. Again, Tukey’s post hoc pairwise comparison revealed that judgments of *Promise* were significantly more favorable than all other scenarios: *Heinz*, *p* < .001, *Dog*, *p* < .001. *Trolley*, *p* < .001; and judgments of *Dog* were significantly harsher than both *Heinz*, *p* = .002 and *Trolley*, *p* = .002; all there were no significant differences in ratings of *Heinz* and *Trolley*, *p* = 1.000.

#### Measuring dumbfounding

**Fig. 4:**
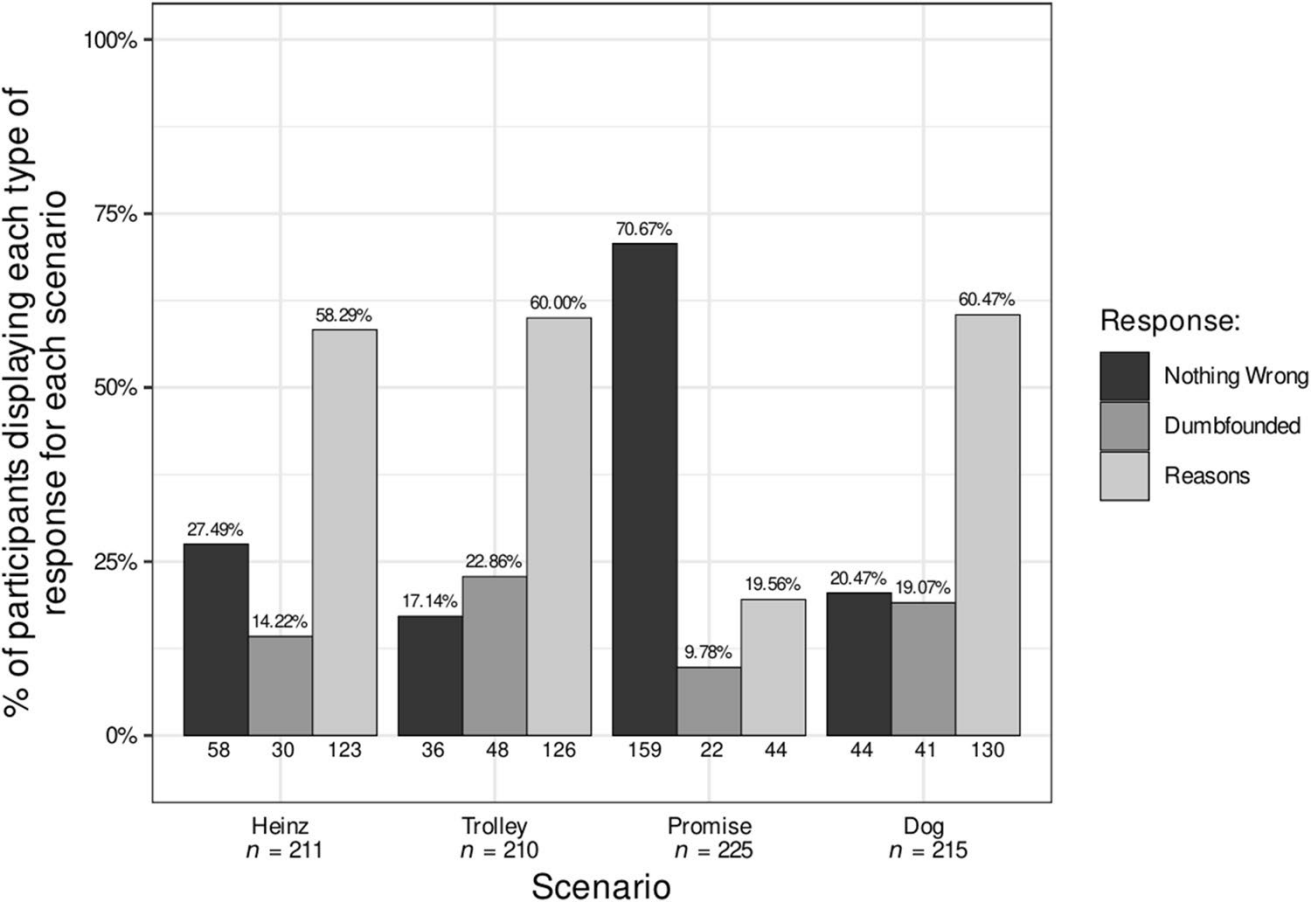
Rates of each type of response for each scenario in the mixed sample

Participants who selected the admission of not having reasons were identified as dumbfounded. Across the four scenarios 99 participants (39.13%) provided a dumbfounded response at least once. Table 4 and Fig. 4 show the number and percentage of participants who selected each response for each scenario. Rates of dumbfounded responding for each scenario in Study 3 were significantly greater than zero, *Heinz*: *z* = 5.68, *p* < .001; *Trolley*: *z* = 7.36, *p* < .001; *Promise*: *z* = 4.81, *p* < .001; *Dog*: *z* = 6.68, *p* < .001.

A chi-square test for independence revealed significant differences in responses to the critical slide depending on which scenario was being discussed, χ^2^(6, *N* = 253) = 186.20, *p* < .001. Table 7 shows the observed counts, expected counts and standardized residuals for each response for each scenario. For *Heinz*, *Dog*, and *Trolley*, people were significantly more likely to provide reasons and less likely to select “there is nothing wrong” than expected by chance. In contrast, for *Promise* participants were more likely to select “there is nothing wrong” than to present as dumbfounded, or to present as dumbfounded or provide reasons. It is possible that finding may be confounded by the responses to *Promise*, however, when *Promise* is excluded from the analysis, the result holds, χ^2^(4, *N* = 253) = 9.73, *p* < .001. Again, we tested for the possibility of order effects, and found no significant differences in responses to the critical slide depending on order of presentation χ^2^(6, *N* = 271) = 3.92, *p* = .687.

**Table 7:** Observed counts, expected counts, and standardized residuals for each response to the critical slide depending on scenario

Response		Dog	Heinz	Promise	Trolley
Nothing Wrong	Observed count	44	58	159	36
	Expected count	74	73	78	72
	Standardized residuals	−5**	−2.46*	13.28**	−6.08**
Dumbfounded	Observed count	41	30	22	48
	Expected count	35	35	37	34
	Standardized residuals	1.23	−0.98	−3.11*	2.92*
Reason	Observed count	130	123	44	126
	Expected count	106	104	111	103
	Standardized residuals	3.84**	3.06*	−10.32**	3.62**

*p < .05. **p < .001

#### Individual differences

A hierarchical linear regression was conducted to test the possible relationship between ICS (Triandis & Gelfand, 2011), MLQ (Steger et al., 2008), and susceptibility to dumbfounding. As in Studies 1 and 2, susceptibility to dumbfounding was operationalized by calculating the number of times a participant provided a dumbfounded response. With this measure as the outcome variable, we included the four subscales of ICS, along with both subscales of the MLQ, as predictor variables in a multinomial logistic regression model. The overall model did not predict susceptibility to dumbfounding, *R*^2^ = .06, *F*(6, 168) –1.90, *p* = .084, in Study 3.

We conducted a series of multinomial logistic regressions to investigate the possible relationship between ICS (Triandis & Gelfand, 2011) and MLQ (Steger et al., 2008), and responding to each of the scenarios individually. Response to the critical slide scenario was the dependent variable for each scenario, and the four subscales of the ICS, along with the two subscales of the MLQ were included as predictor variables.

The overall model predicted responses for the *Heinz* dilemma, χ^2^(12, *N* = 194) = 23.04, *p* = .027, the observed power was 0.92, explaining between 6.89% (Cox and Snell *R* squared) and 10.13% (Nagelkerke’s *R* squared) of the variance in responses to the critical slide. The only significant predictors in the model were Vertical Individualism, and MLQ: Search. As VI increased, participants were significantly more likely to select “there is nothing wrong” than to provide reasons for their judgment, Wald = 7.06, *p* = .438, odds ratio = 0.98, 95% CI [1.02, 1.14]. As MLQ: Search increased, participants were significantly more likely to present as dumbfounded than to provide reasons, Wald = 3.96, *p* = .653, odds ratio = 1.02, 95% CI [1.00, 1.16].

The overall models did not significantly predict responses for any of the other scenarios: *Trolley*, χ^2^(12, *N* = 184) = 11.98, *p* = .447, the observed power was 0.60; *Promise*, χ^2^(12, *N* = 184) = 20.35, *p* = .061, the observed power was 0.87; *Dog*, χ^2^(12, *N* = 184) = 9.27, *p* = .680, the observed power was 0.47.

## General discussion

The primary aim of the current research was to investigate if moral dumbfounding could be evoked in non-Western samples. Across three studies, using standardized materials and procedure (McHugh et al., 2017), we found evidence for dumbfounding in a Chinese sample (Study 1), an Indian sample (Study 2), and a mixed sample, primarily from MENA region (Study 3). This was demonstrated across a range of moral scenarios. Our findings make an important empirical contribution, providing further evidence for the existence of moral dumbfounding, and demonstrating its occurrence in previously unstudied populations.

These studies also make an important theoretical contribution. First, our findings present a further challenge to theorists who argue against the existence of moral dumbfounding (e.g., Gray et al., 2014; Royzman et al., 2015; Schein & Gray, 2018; Wielenberg, 2014). That is, our results pose a challenge to attempts to explain moral judgments as a process of applying moral principles (e.g., Royzman et al., 2015). Regarding the harm principle specifically, while it is possible that our results may plausibly be interpreted as consistent with implicit perceptions of harm (e.g., Gray et al., 2014); previous research has directly tested the role of the harm principle in dumbfounded responding and demonstrated that it cannot adequately explain dumbfounded responding (McHugh et al., 2020; see also Royzman & Borislow, 2022, for demonstration of the inconsistency with which the harm principle is applied). Some “softer” interpretations of dyadic morality exist in the literature suggesting that the harm principle explains the majority, but not necessarily all, of morality (e.g., Gray et al., 2012; Schein & Gray, 2018). While the findings presented here are consistent with this interpretation, it remains unclear how they would be explained by harm-based/dyadic morality approaches.

Second, our findings are consistent with the predictions a categorization approach to moral judgment (MJAC; McHugh et al., 2022). MJAC predicts that dumbfounding can be observed for a broader, more diverse range of scenarios (previous research has primarily focused on the incest taboo, e.g., McHugh et al., 2020; Royzman et al., 2015). Overall, rates of dumbfounded responding were relatively low, however they were in line with existing published work (McHugh et al., 2017, 2020). Low rates of dumbfounded responding are not surprising given the role of accountability demands in decision making (e.g., Tetlock, 2002), that is, people generally appear motivated to be seen to be able to justify their decisions, thus admitting to not having reasons for a judgment is likely an aversive response. Evidence for this aversion to providing a dumbfounded response can be found in the analysis of the interview data presented by McHugh et al. (2017), whereby dumbfounded responses were associated with longer periods of silence, expressions of doubt, and more attempts to generate reasons. Furthermore, we note that according to a categorization approach, categorizations that have become emergently stable (skilled) often mirror “real-world” categories such as natural kinds or social norms (see McHugh et al., 2022, p. 134). In such cases, individuals may be able to draw on their knowledge of these “real-world” categories to formulate plausible justifications for their judgment, thus reducing observed rates of dumbfounded responding. We note that these justifications may be better described as post hoc rationalizations rather than being representative of considerations underlying the initial categorizations (e.g., Haidt, 2001; McHugh et al., 2022; Nisbett & Wilson, 1977; see also Jefferson, 2020). The relatively low rates of observed dumbfounding suggests that people are generally skilled at this rationalization process, however despite these low rates, we did find evidence for dumbfounded responding for each of the scenarios used, with almost half of all participants (*n* = 256 out of a total *N* = 513) provided a dumbfounded response at least once. The presence of dumbfounded responding for a range of moral scenarios in the studies presented here is in line with the prediction of the categorization approach. Furthermore, MJAC predicts that dumbfounding should not be unique to specific populations. The findings presented here also support this prediction. We found evidence for dumbfounding across three diverse, and previously understudied samples; and across six different scenarios.

### Variability and individual differences

Across all three studies we found significant variability depending on scenario. Interestingly, the pattern of the observed variability appeared to vary with country, e.g., for both the Indian sample and the MENA sample, *Trolley* appeared to evoke the highest rates of dumbfounding, while for the Chinese sample, *Cannibal* evoked the highest rates of dumbfounding. In contrast for WEIRD samples, *Incest* tends to be the scenario that most reliably evokes dumbfounding (McHugh et al., 2017).

We explored the possible relationship between dumbfounded responding and individualism/collectivism (Renzhi et al., 2013; Triandis & Gelfand, 2011, Studies 1, 2, and 3) and Meaning in Life: Presence and Search (Steger et al., 2008, Studies 2 and 3). Only in Study 2 were these measures associated with overall susceptibility to dumbfounded responding, with Vertical Individualism negatively predicting dumbfounded responding. Analysis of the items (see Appendix E) suggests this subscale may be providing a measure of people’s motivations to do well or to succeed, therefore viewing dumbfounding as a failure (to provide reasons) may explain this relationship. For *Incest*, Study 2 found that Horizontal Individualism predicted selecting “there is nothing wrong,” while Vertical Collectivism predicted dumbfounded responding*.* Furthermore Meaning in Life: Presence predicted giving reasons for *Cannibal*. In Study 3, for *Heinz*, Vertical Individualism predicted selecting “there is nothing wrong” and MLQ: Search predicted dumbfounded responding. These diverse findings may point to differences in the way particular issues are valued in different cultures, and further study of this cultural variability is warranted.

### Limitations and future directions

A key limitation is that all participants were recruited either through a university (Studies 1, and 3), or were university graduates (Study 2), and participants in both Studies 2 and 3 were proficient in English. As such, the recruited samples are not necessarily representative of their respective populations. Additionally, the links to universities pose a significant challenge to our stated aim of investigating the phenomenon outside of WEIRD or Western populations. We note that In Study 1 all participants were both native to China, and residing in China at the time of study; in Study 2, all participants were native to India, and over 87% (158/181) were residing in India at the time of study; in Study 3 we recorded nationality/country of origin only, and given the data collection method (snowball sampling in an international university), it is likely that participants were not residing in their country of origin. Despite the limitations regarding our samples, moral dumbfounding has not previously been studied in these populations; thus, the present work contributes to scholarship by providing evidence for dumbfounding within a subset of these understudied populations. Future research should investigate the generalizability of these findings with more representative samples.

Another limitation of the current research is the overall relatively low rates of dumbfounding observed (between 9% and 36% depending on scenario and study). Across all studies, and all scenarios, a clear majority of participants indicated that they could provide reasons for their judgments. However, the patterns of results observed here are comparable with similar work using this same measure of dumbfounding (11%–25%, McHugh et al., 2017, Study 3; 17%–19% for the Incest dilemma, McHugh et al., 2020). We note that despite the overall low incidence of dumbfounding, we did find evidence of dumbfounded responding. Thus, our results are consistent with the predictions of a categorization approach (McHugh et al., 2022), and pose a challenge to harm-based or principle-based approaches (Royzman et al., 2015; Schein & Gray, 2018). Future research should investigate the mechanisms that lead to dumbfounding.

Additionally, it could be argued that measuring dumbfounding using the critical slide may not provide a true estimate of the prevalence of dumbfounding. The response options offer a forced choice that may not reflect the experience of all participants, and it may be possible that participants experiencing doubt may select the dumbfounded response because they lack confidence in their reasons (but that they do have reasons). There are good reasons to suggest this is not the case. Firstly, the dumbfounded response on the critical slide has good face validity as a measure of dumbfounding defined as *maintaining a moral judgment in the absence of supporting reasons*, as it includes an affirmation of the judgment accompanied by an explicit admission of not having reasons. This remains true even in cases where participants initially had reasons but these reasons were defeated by counter-arguments, acknowledging their initial reasons are not adequate, participants may identify alternative reasons, change their judgment, or maintain their judgment while acknowledging they cannot support is with reasons. Secondly, evidence from McHugh et al. (2017) suggests that admitting to not having reasons is an aversive response, and selecting an admission of not having reasons on the critical slide was shown to be the most conservative measure of dumbfounded responding across three studies (including the interview Study 1, McHugh et al., 2017). This is preferable to an overly liberal measure that may lead to false positives, and an over-estimation of the prevalence of dumbfounding. We note that the less conservative measure that included coding participants’ open-ended responses for unsupported declarations or tautological responses still yielded relatively low rates of dumbfounded responding (between 26% and 43% depending on the scenario; McHugh et al., 2017).

The moral foreign language effect (Cipolletti et al., 2016) means that the use of an English language survey in Studies 2 and 3 is another potential limitation. People have been shown to make more utilitarian judgements when moral scenarios are presented in another language (Costa et al., 2014), potentially leading to reduced rates of dumbfounding (in favour of selecting “there is nothing wrong”). However, despite this potential confound, dumbfounded responding was observed for all scenarios in Studies 2 and 3.

Finally, we note that our studies were not intended as a systematic investigation of cultural differences in evaluation of specific moral content (there are other research programs dedicated to this; e.g., Haidt & Joseph, 2008; Narvaez, 2016; Shweder et al., 1997). Rather, we extend an existing paradigm and examine the incidence of moral dumbfounding in three previously unstudied contexts. We provide initial evidence for the existence of moral dumbfounding in a Chinese sample, an Indian sample and a mixed, primarily MENA sample.

## Conclusion

Previous research on moral dumbfounding has exclusively studied WEIRD participants. This poses a challenge to the generalizability of the phenomenon. In three studies, we tested whether or not dumbfounded responding could be elicited in non-Western samples. We found evidence for moral dumbfounding in all three samples. Our findings are consistent with and provide some evidence for, a categorization explanation of moral judgment (e.g., MJAC, McHugh et al., 2022). Future research on dumbfounding may provide unique insights into the cognitive mechanisms that govern moral judgments.

## Supplementary Information

ESM 1 (DOCX 36 kb)

## Data Availability

All participant data, and analysis scripts can be found on this paper’s project page on the Open Science Framework (https://osf.io/2h3k7/?view_only=0e36ba29fd3c453f9ae6507b0ebbb8fc). We used R (Version 4.0.3; R Core Team, 2020) for all analyses. See OSF page for full list and full citation for all packages used.
