# Supplementary material for: Just wrong? Or just WEIRD? Investigating the prevalence of moral dumbfounding in non-Western samples
Source: Mem Cognit. 2023 Jan 17;51(5):1043–60. doi: 10.3758/s13421-022-01386-z (PMC10284959; doi:10.3758/s13421-022-01386-z)
Supplement: Supplementary file 1 — (DOCX 36 kb) [file 13421_2022_1386_MOESM1_ESM.docx]

# Appendices

# Appendix A: Moral Scenarios

## Heinz

In Europe, a woman was near death from a very bad disease, a special kind of cancer. There was one drug that the doctors thought might save her. It was a form of radium for which a druggist was charging ten times what the drug cost him to make. The sick woman’s husband, Heinz, went to everyone he knew to borrow the money, but he could only get together about half of what it cost. He told the druggist that his wife was dying, and asked him to sell it cheaper or let him pay later. But the druggist said, “No, I discovered the drug and I’m going to make money from it.” So, Heinz got desperate and broke into the man’s store to steal the drug for his wife. The druggist had Heinz arrested and charged (McHugh et al., 2017; adapted from Haidt et al., 2000).

## Trolley

A Trolley is hurtling down a track towards five people. It will kill them all on impact. Paul is on a bridge under which it will pass. He can stop it by putting something very heavy in front of it. As it happens, there is a very fat man next to him – Paul’s only way to stop the trolley is to push him over the bridge and onto the track, killing him to save five. Paul decides to push the man (McHugh et al., 2017; adapted from Greene et al., 2001).

## Cannibal

Jennifer works in a medical school pathology lab as a research assistant. The lab prepares human cadavers that are used to teach medical students about anatomy. The cadavers come from people who had donated their body for the general use of the researchers in the lab. The bodies are normally cremated, however, severed cuts may be disposed of at the discretion of lab researchers, One night Jennifer is leaving the lab when she sees a body that is going to be discarded the next day. Jennifer was a vegetarian, for moral reasons. She thought it was wrong to kill animals for food. But then, when she saw a body about to be cremated, she thought it was irrational to waste perfectly edible meat. So she cut off a piece of flesh, and took it home and cooked it. The person had died recently of a heart attack, and she cooked the meat thoroughly, so there was no risk of disease (McHugh et al., 2017; adapted from Haidt et al., 2000).

## Incest

Julie and Mark, who are brother and sister, are travelling together in France. They are both on summer vacation from college. One night they are staying alone in a cabin near the beach. They decide that it would be interesting and fun if they tried making love. At very least it would be a new experience for each of them. Julie was already taking birth control pills, but Mark uses a condom too, just to be safe. They both enjoy it, but they decide not to do it again. They keep that night as a special secret between them, which makes them feel even closer to each other (McHugh et al., 2017; adapted from Haidt et al., 2000).

## Promise

A woman was dying, and on her deathbed she asked her son (James) to promise that he would visit her grave every week. The son loved his mother very much, so he promised to visit her grave every week. But after the mother died, the son didn’t keep his promise, because he was very busy (Haidt et al., 1993).

## Dog

A family’s dog was killed by a car in front of their house. They had heard that dog meat was delicious, so they cut up the dog’s body and cooked it and ate it for dinner. The dog had died as a result of the the car accident, and they cooked the meat thoroughly, so there was no risk of disease (Haidt et al., 1993).

# Appendix B: Sample Statements to Challenge Judgments

## Heinz/Druggist

- Do you agree that the druggist has to make a living?
- And do you accept that Heinz broke into the druggist’s store?
- And do you accept that he stole from him?

## Trolley

- Do you accept that five people would have died if Paul didn’t push the man?
- And this man is the only way available to stop the trolley? (Paul does not weigh enough)
- Do you agree that in stopping the trolley Paul saved the lives of five people?

## Cannibal

- The body had been donated for research, it was to be discarded the next day. You must agree then that it had obviously fulfilled its purpose?
- Do you accept that the body was already dead?
- And do you accept that there was no risk of disease?

## Incest

- Do you not agree that any concerns regarding reproductive complications are eased by their using of two forms of contraception?
- And do you accept that they are both consenting adults, and that they both consented and enjoyed it?
- And do you concede that nobody else was affected by their actions?

## Promise

- Do you accept that James’ might genuinely have not enough time to visit his mother’s grave every week?
- Do you accept that nobody was harmed by James failing to visit his mother’s grave?
- Do you agree that James should be free to deal with his grief in his own way?

## Dog

- Do you agree that the family had the right to dispose of the body of their dog in a manner that they choose?
- Do you accept that the death of the dog was beyond the control of the family?
- And do you accept that there was no risk of disease?

# Appendix C: Wordings of the Critical Slides

## Heinz

Heinz’ did what he had to do in trying to save his wife’s life!

How can that be wrong?

## Cannibal

Jennifer’s actions did not harm anyone, or negatively affect anyone.

How can there be anything wrong with what she did?

## Incest

Julie and Mark’s actions did not harm anyone, or negatively affect anyone.

How can there be anything wrong with what they did?

## Trolley

Paul’s actions saved the lives of five people!

How can saving lives be wrong?

## Promise

James was only trying to get his own life back to normal after the passing of his mother.

How can there be anything wrong with what he did?

## Dog

The dog was already dead and there was no risk of disease.

How can there be anything wrong with what the family did?

## Response options (randomized order)

There is nothing wrong.

It’s wrong but I can’t think of a reason.

It’s wrong and I can provide a valid reason.

# Appendix D: Post Discussion Questionnaire

| How sure were you about your judgment? | | | | | | |
| --- | --- | --- | --- | --- | --- | --- |
| Not at all |  |  |  |  |  | Extremely sure |
| 1 | 2 | 3 | 4 | 5 | 6 | 7 |

| How much did you change your mind? | | | | | | |
| --- | --- | --- | --- | --- | --- | --- |
| Not at all |  |  |  |  |  | Extremely |
| 1 | 2 | 3 | 4 | 5 | 6 | 7 |

| How confused were you? | | | | | | |
| --- | --- | --- | --- | --- | --- | --- |
| Not at all |  |  |  |  |  | Extremely confused |
| 1 | 2 | 3 | 4 | 5 | 6 | 7 |

| How irritated were you? | | | | | | |
| --- | --- | --- | --- | --- | --- | --- |
| Not at all |  |  |  |  |  | Extremely irritated |
| 1 | 2 | 3 | 4 | 5 | 6 | 7 |

| How much was your judgment based on reason? | | | | | | |
| --- | --- | --- | --- | --- | --- | --- |
| Not at all |  |  |  |  |  | Extremely |
| 1 | 2 | 3 | 4 | 5 | 6 | 7 |

| How much was your judgment based on “gut” feeling? | | | | | | |
| --- | --- | --- | --- | --- | --- | --- |
| Not at all |  |  |  |  |  | Extremely |
| 1 | 2 | 3 | 4 | 5 | 6 | 7 |

# Appendix E: Individualism / Collectivism Scale

## Vertical Collectivism

VC1: Parents and children must stay together as much as possible.

VC2: It is my duty to take care of my family, even when I have to sacrifice what I want.

VC3: Family members should stick together, no matter what sacrifices are required.

VC4: It is important to me that I respect the decisions made by my groups.

## Horizontal Collectivism

HC1: If a coworker gets a prize, I would feel proud.

HC2: The well-being of my coworkers is important to me.

HC3: To me, pleasure is spending time with others.

HC4: I feel good when I cooperate with others.

## Vertical Individualism

VI1: It is important that I do my job better than others.

VI2: Winning is everything.

VI3: Competition is the law of nature.

VI4: When another person does better than I do, I get tense and aroused.

## Horizontal Individualism

HI1: I’d rather depend on myself than others.

HI2: I rely on myself most of the time; I rarely rely on others.

HI3: I often do “my own thing.”

HI4: My personal identity, independent of others, is very important to me.

# Appendix F: Meaning in Life Questionnaire

I understand my life’s meaning. (P)

I am looking for something that makes my life feel meaningful. (S)

I am always looking to find my life’s purpose. (S)

My life has a clear sense of purpose. (P)

I have a good sense of what makes my life meaningful. (P)

I have discovered a satisfying life purpose. (P)

I am always searching for something that makes my life feel significant. (S)

I am seeking a purpose or mission for my life. (S)

My life has no clear purpose. (P)

I am searching for meaning in my life. (S)

# Appendix G: Supplementary Analysis

Testing predictors of dumbfounding, compared to reason-giving. Overall the model significantly predicted response to the critical slide χ^2^(14) = 459.33, *p* < .001 (0 = *reason-giving*, 1 = *dumbfounding*).

Table S1.

Predictors of dumbfounding compared to reason-giving

|  | *B* | *S.E.* | *df* | *t* | *p* |
| --- | --- | --- | --- | --- | --- |
| (Intercept) | 0.91 | 0.12 | 764.14 | 7.47 | < .001 |
| Initial Rating | -0.01 | 0.01 | 997.73 | -1.30 | .192 |
| Revised Rating | 0.03 | 0.01 | 999.74 | 2.82 | .005 |
| Initial Confidence | 0.00 | 0.01 | 1017.11 | -0.42 | .676 |
| Revised Confidence | -0.06 | 0.01 | 1013.53 | -4.81 | < .001 |
| Confused | 0.04 | 0.01 | 1014.62 | 5.31 | < .001 |
| Irritated | 0.00 | 0.01 | 948.11 | 0.43 | .669 |
| Reason-based | -0.09 | 0.01 | 1012.34 | -11.70 | < .001 |
| Gut-based | 0.00 | 0.01 | 923.85 | 0.86 | .393 |
| Vertical Collectivism | 0.03 | 0.01 | 621.26 | 3.16 | .002 |
| Horizontal Collectivism | -0.02 | 0.01 | 603.45 | -1.52 | .130 |
| Vertical Individualism | 0.00 | 0.01 | 582.03 | -0.30 | .761 |
| Horizontal Individualism | -0.01 | 0.01 | 539.68 | -0.82 | .415 |
| MLQ: Presence | 0.00 | 0.00 | 600.19 | 0.48 | .632 |
| MLQ: Search | 0.00 | 0.00 | 595.54 | -0.13 | .899 |

Testing predictors of nothing-wrong, compared to reason-giving. Overall the model significantly predicted response to the critical slide χ^2^(14) = 1,060.27, *p* < .001 (0 = *reason-giving*, 1 = *nothing wrong*).

Table S2.

Predictors of nothing-wrong compared to reason-giving

|  | *B* | *S.E.* | *df* | *t* | *p* |
| --- | --- | --- | --- | --- | --- |
| (Intercept) | 0.17 | 0.09 | 721.42 | 1.88 | .060 |
| Initial Rating | 0.06 | 0.01 | 1179.65 | 8.35 | < .001 |
| Revised Rating | 0.12 | 0.01 | 1182.48 | 16.20 | < .001 |
| Initial Confidence | -0.01 | 0.01 | 1160.58 | -0.89 | .376 |
| Revised Confidence | -0.02 | 0.01 | 1185.56 | -2.34 | .019 |
| Confused | 0.01 | 0.01 | 1157.96 | 1.14 | .160 |
| Irritated | -0.01 | 0.00 | 1034.61 | -2.39 | .017 |
| Reason-based | -0.02 | 0.01 | 1128.12 | -3.86 | < .001 |
| Gut-based | -0.01 | 0.00 | 894.49 | -1.20 | .232 |
| Vertical Collectivism | 0.00 | 0.01 | 494.01 | -0.36 | .718 |
| Horizontal Collectivism | -0.01 | 0.01 | 500.53 | -0.99 | .322 |
| Vertical Individualism | 0.00 | 0.01 | 499.94 | 0.64 | .522 |
| Horizontal Individualism | 0.00 | 0.01 | 526.55 | 0.88 | .379 |
| MLQ: Presence | 0.00 | 0.00 | 517.93 | 0.78 | .437 |
| MLQ: Search | 0.00 | 0.00 | 482.43 | -0.09 | .927 |

Testing predictors of dumbfounding, compared to nothing-wrong. Overall the model significantly predicted response to the critical slide χ^2^(14) = 405.65, *p* < .001 (0 = *nothing wrong*, 1 = *dumbfounding*).

Table S3.

Predictors of nothing-wrong compared to reason-giving

|  | *B* | *S.E.* | *df* | *t* | *p* |
| --- | --- | --- | --- | --- | --- |
| (Intercept) | 0.85 | 0.12 | 374.39 | 7.04 | < .001 |
| Initial Rating | -0.05 | 0.01 | 698.58 | -4.72 | < .001 |
| Revised Rating | -0.09 | 0.01 | 698.41 | -8.05 | < .001 |
| Initial Confidence | 0.02 | 0.01 | 684.55 | 1.35 | .178 |
| Revised Confidence | -0.02 | 0.01 | 686.26 | -1.51 | .132 |
| Confused | 0.01 | 0.01 | 665.85 | 1.36 | .176 |
| Irritated | 0.03 | 0.01 | 622.86 | 4.16 | < .001 |
| Reason-based | -0.03 | 0.01 | 598.88 | -3.67 | < .001 |
| Gut-based | 0.02 | 0.01 | 519.02 | 2.36 | .019 |
| Vertical Collectivism | 0.01 | 0.01 | 311.15 | 1.48 | .139 |
| Horizontal Collectivism | 0.01 | 0.01 | 344.32 | 1.02 | .308 |
| Vertical Individualism | 0.01 | 0.01 | 456.04 | 0.58 | .561 |
| Horizontal Individualism | -0.02 | 0.01 | 348.35 | -2.06 | .040 |
| MLQ: Presence | 0.00 | 0.00 | 389.89 | -1.49 | .136 |
| MLQ: Search | 0.00 | 0.00 | 370.46 | 0.31 | .757 |
